# Get Smart: Learning and partnership with Ethiopia’s Health Extension Programme to re-envision contraceptive service delivery to young couples

**DOI:** 10.12688/gatesopenres.12970.1

**Published:** 2019-10-15

**Authors:** Gabrielle Appleford, Claire Cole, Metsehate Ayenekulu, Sue Newport, Emma Mulhern

**Affiliations:** 1 Itad, Preece House, Davigdor Road, Hove, BN3 1RE, UK; 2 Population Services International, 1120 19th Street Northwest, Suite 600, Washington, DC, 20036, USA; 3 Population Services International, METI Office Park, Bole Sub City, Kebelle 03/07, Namibia Street, Addis Ababa, Ethiopia

**Keywords:** Contraception, adolescent sexual and reproductive health, health extension programme

## Abstract

**Background: **Adolescents 360 (A360) implements the Smart Start (SS) programme through Ethiopia’s Health Extension Programme (HEP). SS is premised on financial planning as an entry point to discuss family planning (FP) with newly married couples and central to its delivery are the health extension workers (HEW). This article evaluates the A360 experience and learning from the process evaluation implemented by Itad to understand contextual barriers and enablers from the perspective of the HEW.

**Methods: **A purposive sampling strategy was employed whereby 27 key stakeholders were identified from Oromia, Addis Ababa and Amhara, based on exposure to the SS programme. Findings from the action research were shared with A360 through a one day sounding workshop.

**Results: **Findings revealed that many local government and communal respondents do not view adolescent pregnancy as a problem, unless out of wedlock, and adolescent pregnancy is closely linked to early marriage. As a result, some providers, including HEWs, acknowledged that married adolescent girls were previously ‘neglected’ by them, while husbands indicated that they had not previously been included in FP counselling. Findings also revealed some challenges with SS implementation as HEWs were ‘deprioritizing’ the intervention and many HEWs had been *in situ* for several years and were overworked and frustrated. Against this backdrop, A360 was viewed as adding to the HEW workload. While the programme design was focused on adolescent users, there was increasing recognition that HEWs also needed to be at the centre of solution design.

**Conclusions: **Despite challenges associated with the HEP, Ethiopia FP2020 plans to support the ‘next generation’ of HEWs, including a focus on adolescents and youth. To gain deeper insight and put the HEW at the centre of design, A360 will continue to work with the process evaluation to understand contextual barriers and enablers from the perspective of the HEW.

## Introduction

*How might we better meet the needs of adolescent couples with contraceptive counselling and services through Ethiopia’s health extension programme (HEP)?*

This was the motivation behind the integration of Adolescents 360 (A360) into Ethiopia’s health extension programme (HEP), resulting in the creation of the Smart Start programme (SS; Box 1). The HEP is largely attributed with a ‘significant and systematic’ increase in the modern contraceptive prevalence rate and health equity improvements ^1^. This includes for married, childbearing adolescents with no or little education, or those living in rural areas. Despite these achievements, low utilisation of and large unmet need for contraception remains among this population, alongside increasing recognition of an overstretched HEP and overworked health extension workers (HEWs), the ‘backbone’ of rural primary health care (PHC).

Box 1. **Smart Start solution**

*“Smart Start focuses on what’s relevant to married adolescent girls: their ability to set up a new home for the first time, to find a path to financial stability, and to have healthy children. With Smart Start, contraception becomes a key tool to achieving these goals.”*

A360 Ethiopia business case

Through a transdisciplinary approach, A360 merges public health, human-centred design (HCD), adolescent developmental science, socio-cultural anthropology, youth engagement and social marketing to yield country-specific adolescent and youth sexual and reproductive health solutions. The A360 project is implemented by Population Services International (PSI) and works in partnership with IDEO.org, Center for the Developing Adolescent at University of California, Berkeley and the Society for Family Health Nigeria. A360 is co-funded by the Bill and Melinda Gates Foundation and the Children’s Investment Fund Foundation.

In the Ethiopian context, the SS programme, premised on financial planning, is an entry point to discuss family planning with newly married couples. Whilst the design of the programme was guided by adolescent girls, central to the delivery of are the HEW. The HEW are supported by a PSI Smart Start Navigator (SSN) to deliver couples counselling and family planning (FP) services (“catch up”) but are subsequently left to continue the SS programme (“keep up”), while the SSN moves to another location. The SS programme has been tasked with demonstrating its effectiveness to the HEP before its adoption by the Federal Ministry of Health (FMoH) of Ethiopia.

In this article, we describe the result of participatory action research (PAR) undertaken to evaluate the A360 project, which includes evaluation of the HEP, the HEW and the SS programme.

## Methods

Itad is working in collaboration with the London School of Hygiene and Tropical Medicine (LSHTM) and Avenir Health to independently evaluate and distil lessons from the A360 project. As part of this evaluation, a process evaluation (PE) has been specifically designed to support A360 with ‘uncovering’ contextual enablers and barriers and to guide adaptive management and course correction. The PE, grounded in A360’s theory of change, has four areas of inquiry - process, context, experience and solution. To date, the PE has included four rounds of data collection over the period of 2016-2017 in two regions and one municipality of Ethiopia; Oromia, Amhara and Addis Ababa.

**Figure 1.**
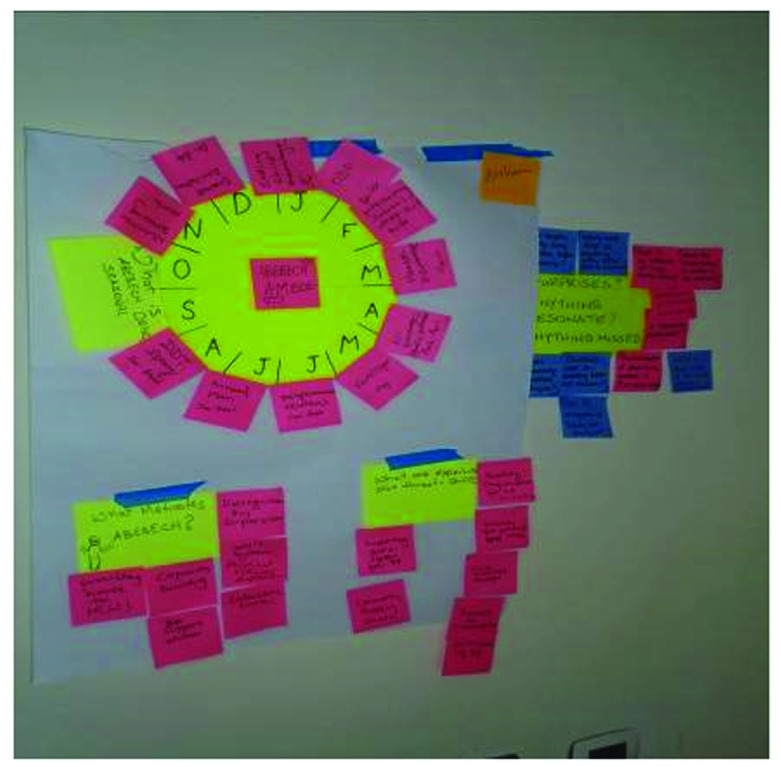
Seasonal calendar prepared during the workshop.

In early 2018, the PE conducted PAR, the basis of the findings presented below ^a^. This involved co-development of the action research focus with A360, while data collection and analysis were conducted independently by a PE team. A purposive sampling strategy was employed whereby, in consultation with the A360 Ethiopia team, key stakeholders were identified, based on exposure to the SS programme. These included Federal Ministry of Health programme managers; HEP staff, including HEWs; and, PSI staff involved in SS programme delivery. Staff were interviewed using discussion guides (see *Extended data*) ^2^ in their places of employment and interviews took approximately 45 minutes to one hour. Participant oral consent was sought in advance of interview. Oral consent was selected due to poor literacy of some participants and potential reluctance to sign documents. These were taped using a digital recorder, de-identified and later transcribed. In total, 27 key stakeholders were interviewed from Oromia, Addis Ababa and Amhara. Findings from the PAR were shared with A360 through a one day sounding workshop facilitated by the evaluation team in a participatory manner. The workshop provided a safe space where team members could critically engage with PE findings and reflect on what this means for A360 learning and optimization of implemented solutions. This included interactive sessions where A360 team members identified areas for further exploration, for example, through the use of a seasonal calendar to reflect on HEW workload and other touch points for introducing family planning as part of household financial planning ( Figure 1).

### Data analysis

**Figure 2.**
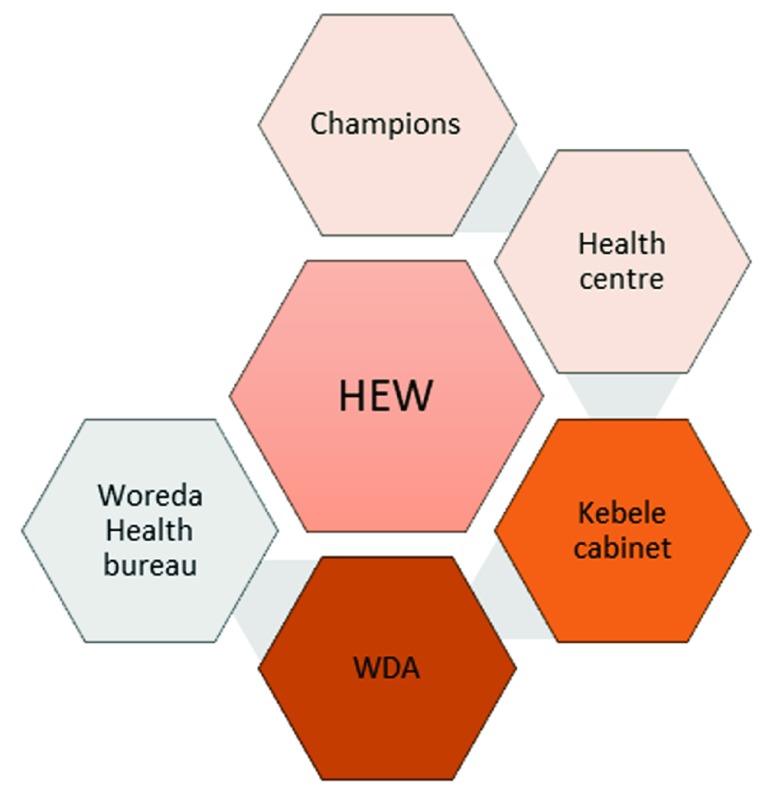
Heat map of community structures.

Analysis was undertaken by the PE team lead and reviewed by the two national PE researchers. Key informant interviews were coded using qualitative analysis software (Dedoose v8.2.12; other non-proprietary software for coding includes QDA Miner Lite). A pre-defined coding frame was employed (see *Extended data*) ^2^, aligned to the domains of the PE and relevant evaluation questions. Common themes were drawn out into a word narrative document (see *Extended data*) ^2^, tracking relative weight of number of respondents expressing a view. This was also used to develop the basic heat map ( Figure 2).

## Key findings

Findings are presented using the three areas of inquiry for the PAR: the HEP; the HEW; and the solution - Smart Start. We present implications at the end of the article to guide further investigation.

### Health Extension Program

From Health Extension Workers (HEWs) through to Ministry of Health (MoH) program managers, there is strong agreement that maternal and child health (MCH) is the priority of the HEP. Family planning is also recognised as a priority but relative to MCH, and, in many instances, is viewed as being *for* mothers. Older and high parity mothers are reported as prioritized. While adolescent health is included in the HEP, this is viewed more as health promotion and less as service delivery.

*“…the [health extension] program has a problem in addressing health access for adolescents.”* MoH Respondent

For the HEP, the intersection of FP and adolescents is MCH, when an adolescent girl becomes pregnant. Ethiopia has a high teenage pregnancy rate (18% of all women giving birth in rural areas) and high rates of child marriage remain prevalent in all regions (49% in rural areas) ^3^. The MoH has made both a key priority and has defined a national adolescent and youth health strategy ^3^. To avoid teenage pregnancy, if not child marriage, some respondents noted that mothers sometimes bring their daughters for family planning in *advance* of marriage.

*“…the [health extension] program has a problem in addressing health access for adolescents.”* MoH Respondent

### Health Extension Worker

HEWs reported an extensive list of tasks that they perform, with most citing MCH activities as their priority. In most instances, HEWs expressed satisfaction in making children well, and saving mothers’ lives. Satisfaction was reported to come from seeing a visible change, such as a heathy child, a clean environment or a happy mother.

*“If you are a mother I think you would understand what a mother feels when her child gets sick and what she feels when he gets better. So, I am very satisfied when I see a mother’s happy face.”* HEW

Similar to MoH respondents, HEWs noted seasonal variation in their priorities and workload. These varied by region, informed by HEP plans and priorities and influenced by topography and demography.

***Dry season.*** Workload increases during the dry season as the HEWs do outreach to households.

*“The mode of life our community is mobile so it is difficult to access community during dry seasons. However, HEWs provide family planning services to the community either before they leave or where they settle through outreach programs. And thus, workload increases during dry season for we have frequent outreaches.”* MoH Respondent

***Rainy season.*** The rainy season is associated with discontinuation of short-term FP methods and unintended pregnancy. While FP does not have a ‘season’, adoption and continuation are seasonally impacted and is one of the main reasons that the MoH is promoting long-acting reversible contraception (LARC).

*“During rainy seasons the work load became less because it is very challenging to come to the health post due to the over flow of water. Even the number of pregnancies increase during the rainy seasons since most women discontinue using family planning.”* (HEW Respondent)

***Wedding season.*** While the SS programme has identified the wedding season ^b^ as key months for promoting adolescent contraception, weddings are associated with demonstrating fertility. Some respondents further acknowledged that adolescents have already engaged in sex before the wedding and this is ‘just the formality’.

*“The official wedding is just for the formality, I can say most of them are already start sex or they are living together before the wedding. Some of the adolescents are pregnant on their wedding.”* HEW

Adolescents without children have not been the main ‘target audience’ of HEWs. Smart Start is associated with having changed this for married adolescents but this may not be the case for unmarried adolescents.

*“So, if they use family planning, they get time to think about their future life. Early marriage is common in our surrounding*.” HEW

Through the sounding workshop, participants developed seasonal calendars for Oromia and Amhara regions. They identified that HEWs performed a number of non-health related activities that involve husbands, such as seasonal distribution of fertiliser, suggesting that there may be other touch points for introducing family planning as part of household financial planning.

### Smart Start programme

Financial planning for couples was viewed as part of Smart Start’s unique selling point (USP). The counselling manual (see *Extended data*) ^2^, produced for this programme, is considered attractive and the images engaging for participants and HEWs. While the manual appeared to generate dialogue with couples sometimes the HEW cannot address their questions.

*“These manuals make Smart Start program out of the ordinary.”* HEW

Despite the manual’s attraction, counselling with it is reported to take a long time (approximately one hour). While HEWs reported that they were comfortable with counselling on financial planning, where Smart Start Navigators (SSNs) have been working, the division of labour has seen SSNs provide the financial counselling while HEWs focus on FP. This is likely due to HEW workload and greater comfort with FP.

*“It takes you more than 30 minutes in order to bring a mother to family planning. So, the short hand of this manual would be important. The current manual differs from the previous manuals that it focuses more on economic aspect while the previous ones focuses on explaining about the medicines*.” HEW

Couples counselling is also viewed as part of SS’s ‘USP’. While this resonated with MoH respondents, in practice it was difficult to implement. A husband’s lack of availability featured as part of the challenge. The heavy workload of married adolescent girls was also reported to constrain their availability.

*“In the absence of husbands, we provide counselling to the wives and ask them to come back with their husbands. In so doing, we go half a way.”* PSI Ethiopia Respondent

HEWs reported being satisfied when couples agreed to be counselled and took up a FP method. When this was not the case, it was reported as frustrating, given the time taken to visit the household and do the counselling.

*“It is a difficult task but I feel happy when they accept my advice. Contrary to this I feel more tired when they ignore me.”* HEW

### Smart Start support

A360 has introduced SSNs (Oromia only) and *woreda* (district) health advisors (Oromia and Amhara) to support SS implementation. SSNs were viewed as an extra pair of hands, working closely with, and sharing the workload of, HEWs. Smart Start staff based at woreda level troubleshoot supply-side constraints in the health system. HEWs expressed the need for more ‘morale and technical support’ after the SSN has left the *kebele* (village).

*“We support her in SS program and she also support us in other health extension activity. We have very good relationship with her…If they provide the SS counselling to the married adolescents, I can stay in the health post to perform other health extension activities.”* HEW

HEW support for SS has ranged from resistance, to bargaining, to acceptance. Resistance was reported initially at the time of the SS training and mainly emanated from HEW concerns about workload. Bargaining is also evident in the way some HEWs have agreed a division of labour with SSNs, whereby HEWs focus on family planning while SSNs focus on the financial counselling. Finally, in some instances, there is acceptance, underpinned by an intrinsic motivation to address adolescents’ wellbeing.

*“On the training we were resistant to accept and implement SS program, we mentioned that we are very busy, and we have very much work loaded in other HEP, but later we believed that it is our responsibility to serve the community... I will consider Smart Start as part of my routine job so that I can get mental satisfaction, I will do it not to be judged by my conscious.”* HEW

The community plays a critical but under recognised role (in terms of support) for the SS programme. In particular, kebele and women’s development army (WDA) leaders were considered as playing a brokerage role within their communities. In many instances, the WDA actively supports the identification of newly married couples and mediates with husbands to get them to attend counselling.

*“We should empower the community, particularly the grassroots level stakeholders, for example, women groups in terms of skills, attitudes, so that the community produce its own health.”* MoH Respondent

### Smart Start future

There are a number of potential sources of support for Smart Start as it looks to the future and optimization.

***Community leading.*** There was opinion that the community does not need to be pursued by SS, but rather should be the pursuers of the programme. This suggests that rather than ‘pushing’ SS through door-to-door visits, SS should ‘pull’ the community using existing community structures and natural touch points. Using existing community structures may make it easier to engage with husbands, considered a USP of the Smart Start solution. In particular, the kebele leadership was viewed as highly influential with husbands if they themselves are convinced. Not to be under-estimated, WDA leaders were also viewed as being able to convince husbands about the merits of Smart Start. A rough heat map ^c^ of the importance of community structures is included in Figure 2 based upon respondent feedback.

*“Once awareness is created effectively the community asks the service by itself”* MoH Respondent.
*“More influential for them [husbands] is the Kebele leader. So, if we use the Kebele leader to talk to the husband, it would be better and more influential.”* MoH Respondent

***Entry point.*** If HEWs can integrate financial planning into FP, can FP be included in financial planning? Using a broader community structure, and being led by the community, may open more possibilities for integration and leverage. There is precedent for this within resilience programmes which the SS programme may wish to explore ^d^.

*“In our community when people meet they ask each other about their health, finances or businesses next to greeting. Therefore, there is no problem in discussing financial planning and then family planning simultaneously.”* MoH Respondent
*“The kebele administration structure is supposed to work on transforming the livelihood of households. Part of that is family spacing and working on a planned manner…”* MoH Respondent

***Health system.*** Smart Start requires greater integration into the health system if it is to be absorbed into the HEP. Greater integration may facilitate adoption in other kebeles (within clusters) without the need for additional Smart Start human resources, as is currently the case. With communities leading and more communal touch points for Smart Start, the solution may be less vulnerable to weaknesses in the health system. At the moment, Smart Start is highly reliant on the HEW, their role being seen as ‘decisive’ to its future.

*“It may continue in some kebeles. You cannot predict at this early stage. It depends on what HEWs do mainly with regards to finding and convincing the newly married couple to bring them to family planning services. In other words, the role of HEWs is decisive.”* MoH Respondent

## Conclusion and implications

Despite challenges associated with the HEP, Ethiopia FP2020 plans to support the ‘next generation’ of HEWs and ensure LARC capacity at PHC levels. This includes a focus on adolescents and youth. The A360 experience and learning from the Itad PE can be brought to bear on this ‘next-gen’ aspiration. To gain deeper insight and put the HEW at the centre of design, A360 will work with the PE to understand contextual barriers and enablers from the ** perspective of the HEW.

Based on the findings from this action research and the sounding workshop, PSI Ethiopia will work with IDEO.org to explore a number of questions through a HCD process, with the HEW at the centre. As such, the conclusions are framed as questions in order to guide the HCD process. These may also have wider application for the HEP.

- Should Smart Start expand its focus from recently married adolescents, to those who have been married for some time and have had a child/children already? Should Smart Start also address unmarried adolescents given that they may already be having pre-marital sex?
- Seasons matter to the HEW workload. How can we plan around seasonal variation to optimise Smart Start and make it easier for HEWs to perform their tasks?
- How to position FP relative to MCH - what brings HEWs job satisfaction? Is it found in the absence of an event, such as an unplanned pregnancy?
- How can Smart Start capitalize upon its USP of engaging men in family planning through financial planning? Are there other touch points with men that Smart Start can mobilise?
- What motivates mothers to bring their daughters for family planning in advance of marriage? How can we learn from these early adopters without revealing their secret?
- What is the cost-benefit of Smart Start to the MoH? How can both integration into the HEP and cost effectiveness be improved?
- What is the cost-benefit of Smart Start to communities? How can both integration into other community-based activities and cost effectiveness be improved? How can communities ‘pull’ on Smart Start, so that is requires less ‘push’ from A360?

## Data availability

### Underlying data

The underlying data for this study consisted of interview transcripts which were coded and analysed and sounding workshop minutes. Approval was obtained from participants to use their anonymised data within research analysis and reports. However, the raw data transcripts contain a number of references to locations, organisations and individuals, which means that they cannot be effectively de-identified. Therefore, in order to protect participant privacy, transcripts and minutes have not been made available in a public repository. In order to request access to these files, please contact mary.lagaay@itad.com stating the reason access is being requested. Approval will be granted on a case-by-case basis (e.g. to researchers for specific research purposes), subject to ethics approval.

### Extended data

Open Science Framework: A360 Ethiopia supporting documents. https://doi.org/10.17605/OSF.IO/E5AVF ^2^

This project contains the following extended data:

- A360 Ethiopia Case Study Data Collection Guide_V4_2018.04.20.doc (Data collection guide including discussion guides, consent form and write-up templates)
- Discussion Aide_HEW_Short_Amharic.pdf (Smart Start counselling manual)
- ET HEW case study_Proofread.docx (Full case study document)
- ET coding framework PDF.pdf (Coding framework used for data analysis)
- ET Analysis_Narrative.pdf (Narrative document prepared during analysis)

Data are available under the terms of the Creative Commons Attribution 4.0 International license (CC-BY 4.0).

## Footnotes

^a^Participatory action research is “a set of principles and practices for originating, designing, conducting, analysing and acting on a piece of research... Within PAR projects, many different methods may be used.” ( http://www.aral.com.au/resources/guide.html).

^b^The months of January and April are wedding ‘seasons’ for Christians, there is no season for Muslims.

^c^Where the intensity of the colour on the heat scale (blue/cool through to red/hot) is indicative of the level of importance of the community structures

^d^ **Partnership with a Microfinance Organization:** In India, PROGRESS partnered with the Network of Entrepreneurship & Economic Development (NEED) to test an intervention through which village health guides provided family planning messages and referrals. **Family Planning Services through Agricultural Field Days:** Pilot research to integrate health services into field days sponsored by Land O’Lakes dairy cooperatives in Kenya successfully linked underserved populations with health services, including resupply of family planning methods. **Partnership with the Green Belt Movement:** Staff of the Greenbelt Movement's population, health and environment project in Kenya were trained to teach volunteers how to integrate family planning discussions into their routine environmental activities.
