# Supplementary material for: Get Smart: Learning and partnership with Ethiopia’s Health Extension Programme to re-envision contraceptive service delivery to young couples
Source: Gates Open Res. 2019 Oct 15;3:1570. [Version 1] doi: 10.12688/gatesopenres.12970.1 (PMC7005467; doi:10.12688/gatesopenres.12970.1)
Supplement: Peer review history

# Peer review: Reviewer response for version 1

**DOI:** 10.21956/gatesopenres.14077.r28348

**Published:** 2020-02-06

**Authors:** Kate Sabot

**Affiliations:** 1 The Centre for Maternal, Adolescent, Reproductive and Child Health (MARCH), London School of Hygiene and Tropical Medicine, London, UK

**Summary:**

 This paper describes a process evaluation of the Strong Start (SS) component of the Adolescent360 (A360) adolescent family planning program implemented by Population Services International (PSI) in select geographies within Ethiopia. The program was designed using HCD methods with adolescent women to ensure a user-centric approach to improving uptake of family planning among a hard to reach demographic. The SS program uses financial planning as an entry point to initiate family planning counselling sessions with newly married adolescent couples relying upon the Ministry of Health’s Health Extension Program (HEP) platform to deliver this service. The cornerstone of the HEP are the Health Extension Workers (HEW), who along with assistance from Strong Start Navigators (SSN) in one region (Oromia), conducted approximately one-hour long counselling sessions using a visual discussion guide.

 The process evaluation involved in-depth interviews (IDI) with 27 stakeholders drawn from the MOH federal/national, regional and zonal leadership, PSI staff, primary health care unit (PHCU) staff, HEWs and SSNs. These interviews were then analyzed and the findings summarized with recommendations for the next phase of implementation. While the discussion guides for the IDIs cover a wide range of topics, the paper focuses on the most actionable findings from the process evaluation, chief among them to shift the focus from adolescents to the health extension workers themselves, to ensure the program design is reflective of HEW perspective, given the model is reliant on them to drive implementation. The innovative and iterative approach to program development and the value of the richly qualitative process evaluation is important to share with the broader research community. 

 Methodologically sound and well written, my primary suggestion is to further situate the SS program and the PE findings in the context of the existing literature, as that will mark the difference between this being a rigorous case study and a research paper. It would also be helpful to have a little more detail regarding the SS program.

 In my report assessments I responded “partly” to two questions. Below are further notes as to why. I then provide further comments and by paper section below. 

 It was a delight to read this paper, I congratulate the authors for sharing their process and findings with the academic research community, as applied/implementation research studies often de-prioritize publication in the interest of focusing efforts and attention on translation of findings into programmatic improvements. There is great value in scholarly analyses of implementation research for both informing and sharing best public health practices and ensuring the relevance of academic research. 

 I hope my comments are helpful. 

**“Is the work clearly and accurately presented and does it cite the current literature?” Partly**

 The paper would benefit from referencing in the introduction articles covering background on the Health Extension Program, family planning models like this one in other contexts and in the discussion, grounding the interpretation of the findings in existing literature evaluating the HEPs. See below for more details in the respective sections of the paper. 

**“Are sufficient details of methods and analysis provided to allow replication by others?” Partly**

 In reviewing the COREQ checklist one area was not provided: non-participation. Consider including if/how many potential participants refused and the reasons for refusal, if known. Additionally, the FMOH discussion guide was missing (see below under supporting documentation for more details).

**Comments on paper sections:**

**Abstract:**

 The methods subsection specifies the number of participants but the method of data collection is missing. While this is provided in the full text, the abstract should include this. It also was not explicit if the sounding workshop was a method of data collection/validation or purely dissemination of research findings the way it is described in the abstract.     

**Introduction:**

 The introduction would benefit from a brief description of the history, structure and scope of the HEP and the Ethiopian Health system, referenced accordingly. Details such as how long it has been in existence, who is involved (HEWs, WDA, PHCUs) and the services provided (even just the number and how that has changed over time to convey scope well beyond MCH and one that has been growing substantially over time) would provide helpful context to interpreting some of the findings. Also,“WDA” appears in the heat map in Figure 2 before the term is defined, readers who are unfamiliar with the Ethiopian health system may not be a little lost without further explanation of how they relate to the HEWs/HEP.

 A paragraph that describes the SS program would be useful. Specifically describing what was implemented (2 day training to X HEWs/ X per PHCU, for example or X HEWs within a selection of X PHCUs) when it was implemented, and at what scale would be useful. While the process evaluation timeline was clear, it wasn’t entirely clear the timeframe of the A360 or SS program implementation. It was not clear how the SSNs relate to the WDA (if they were selected from the WDA, who are intended to support HEWs) or if they were a new and separate structure. It would be helpful to know how the SSNs were selected. If I missed this description of the SS, my apologies! If there isn’t space to go into details around the SS/A360, these descriptions could be placed in supporting documentation.

 While the focus of the PE is inherently qualitative, are there any quantitative programmatic monitoring results that could be reported? Number of HEWs trained, uptake of the SS program? I.e. couples counselled? Uptake of FP among those counselled?

 It would be helpful context to understand if, how and when the FMOH was engaged in the design of SS. From reading the article it was not immediately apparent if they were involved in the design, or just in the discussions around the threshold of evidence required for scale up. 

**Methods:**

 I would recommend defining what a “sounding workshop” means in this context.

 It would also be helpful to understand non-participation — how many refused and the reasons for dropping out, if known?   

**Findings:**

 “Ethiopia has a high teenage pregnancy rate (18% of all women giving birth in rural areas) and high rates of child marriage remain prevalent in all regions (49% in rural areas)”.  The reference provided for these statistics should be checked, I think it may have just been an error as the subsequent reference was to the National Strategy and the citation is listed as the same. The stats should reference the original source, which is unlikely to be the national strategy itself. There may be a relevant updated stat to cite from the 2019 miniDHS, although I have not confirmed if these data points specifically are available. 

 There were questions in the discussion guide regarding other extension service providers and the opportunities for integration. Was the agriculture extension program discussed? The HEP was modelled after the agriculture equivalent and it primarily engages men. Given the recommendation to explore other touch points with men for financial planning and family planning integration, I would have thought this might have come up in the interviews. 

 Since this is a research paper I would expect a brief discussion of how the findings are supported by or refute existing literature both in Ethiopia and more broadly. For example, one finding was the challenge HEWs have with their existing workload. There are a number of articles that explore the workload of HEWs (time motion series study among others) and how that has changed over time, and hence grounding the findings that those HEWs who have been in situ for longer were frustrated by the additional work presented by implementing the SS. 

**Supporting documentation:**

**“ET HEW case study_Proofread.docx (Version: 1)”: **This appears to be highly repetitive with the paper itself. I would suggest removing the repetitive content and only including that which is additional to the paper. Furthermore, this appears to be a draft as it includes notes to a graphic designer and typos (see first spelling of mCPR).

**A360 Ethiopia Case Study_Data Collection Guide_V4_2018.04.20.doc (Version: 1):** It is really helpful to see the field guide, not all papers include such a level of detail. However, I did notice that the interview guide for the FMOH is not included, as it appears it was to be added after a briefing with PSI. Perhaps this is not the latest version? If the document was ultimately not updated, as happens, perhaps the FMOH discussion guide that was used could be included as a separate supporting documentation?

I confirm that I have read this submission and believe that I have an appropriate level of expertise to confirm that it is of an acceptable scientific standard.

# Peer review: Reviewer response for version 1

**DOI:** 10.21956/gatesopenres.14077.r28228

**Published:** 2019-11-25

**Authors:** Beth Anne Pratt

**Affiliations:** 1 Global Health Insights, New York, NY, USA

This paper is a report on the findings of a process evaluation of the Adolescents 360 (A360) project in Ethiopia which targeted health extension workers (HEWs) in the delivery of adolescent-friendly reproductive health interventions. In particular, it focuses on the Smart Start component of the project which involves introducing financial planning into the counselling of newly married couples as a gateway to improving family planning (FP) uptake. The paper explores findings of and lessons from the process evaluation and proposes a number of solutions to overcome the implementation and contextual barriers identified. The paper concludes by raising a number of issues with respect to the design of Smart Start going forward (e.g. that demand and ownership by the community should be improved by shifting from a ‘push’ to a ‘pull’ system of community engagement; that the project might benefit from making the family planning-financial planning connection a bit more of a two-way street; and that Smart Start is still mostly an external project that is not really integrated into the routine of Ethiopia’s Health Extension Program (HEP) within which HEWs work).

 The methodology was based on participatory action research (PAR) and was entirely qualitative, and thus statistical analysis was not applicable to the findings. The qualitative nature of the article is its strength, as the perspectives of the HEWs with respect to the purpose and implementation of Smart Start (i.e. that adolescent pregnancy is not a problem; that Smart Start might increase HEWs already overwhelming workload; and that the health extension project as a platform for Smart Start itself already has a number of issues with respect to prioritization of adolescents) are important health systems and socio-cultural lenses as to the potential hurdles that A360 and Smart Start must overcome. These sorts of issues are often not touched upon by evaluations which frequently focus on whether projects succeed in achieving objectives and targets and treat context as something simply mentioned in passing. The uniqueness of the article was in how it made health systems context and cultural context absolutely central. 

 The only real recommendation I would have with respect to strengthening the article would be to situate it a bit more in existing literature on community health workers, their use as program implementers, and the strengths and weaknesses of their involvement with respect project implementation. Just a couple of paragraphs on what has been written about this issue, first, elsewhere (I think there is quite a lot of literature, for example, on this issue from Nepal, Pakistan, and India, as well as a number of review articles) and, then, with respect to Ethiopia specifically (I think too there is quite a lot on HEWs and barriers to implementation) – Maryse Kok would be someone one would probably wish to cite at some point - and then a sentence on what the research contributes to this body of literature would strengthen the paper. Otherwise, I really enjoyed reading it and recommend it to be indexed.

I confirm that I have read this submission and believe that I have an appropriate level of expertise to confirm that it is of an acceptable scientific standard.
